# The influence of light and heavy training weeks on the cortisol and testosterone awakening responses of elite male judokas: is skeletal muscle damage a mediating factor?

**DOI:** 10.5114/biolsport.2024.135415

**Published:** 2024-04-25

**Authors:** Blair T. Crewther, Benjamin G. Serpell, Christian J. Cook, Zbigniew Obmiński

**Affiliations:** 1 School of Science and Technology, University of New England, Armidale, Australia; 2 Institute of Sport – National Research Institute, Warsaw, Poland; 3 School of Electrical Engineering and Robotics, Queensland University of Technology, Brisbane, Australia; 4 Geelong Cats Football Club, Geelong, Victoria, Australia; 5 Hamlyn Centre, Imperial College, London, UK

**Keywords:** Stress, Adaptability, Recovery, Combat Sport, Physical Performance, HPG, HPA

## Abstract

In sport, the awakening responses of cortisol (CAR) and testosterone (TAR) have been used as evaluative tools. Research findings are, however, inconsistent and the mechanisms involved are unclear. This study investigated the CAR and TAR in male athletes across light and heavy training weeks, focusing on skeletal muscle damage as a mediating factor. Twenty elite male judokas were assessed across consecutive weeks of light and heavy training (i.e., 6 days, 9–10 weekly sessions). Plasma cortisol and testosterone concentrations were measured post-awakening (+3, +30, +60 mins), along with creatine kinase (CK) at +3 mins. The CAR and TAR were indexed by baseline-corrected change scores (Δb30, Δb60) and area under the curve (AUCb30, AUCb60). The early-morning surge in plasma cortisol concentration (CAR_Δb30_, CAR_Δb60_, CAR_AUCb30_, CAR_AUCb60_) was significantly larger after light versus heavy training with effect-size differences of 2.14–2.64. The post-awakening decline in plasma testosterone (TAR_Δb30_, TAR_AUCb30_, TAR_AUCb60_) was found to be significantly less pronounced, whilst CK level was elevated, after heavy than light training with effect-size differences of 0.95–1.04 and 4.70, respectively. Causal mediation analysis confirmed that CK mediated, in part, the training effect on the CAR, but not TAR, measures. In summary, male judokas, upon rising after a light training block, displayed a rising CAR (36%, 22%) and declining TAR (-11%, -15%) at +30 and +60 mins. A high-intensity training block suppressed the CAR (21%, 8%) and attenuated the TAR (-7%, -13%) with accompanying muscle damage offering one mechanism to partly explain the CAR differences.

## INTRODUCTION

In sport, the cortisol awakening response (CAR) has garnered interest to evaluate the physical and/or psychological effects of training and competition [1]. The CAR is a distinct and reliable feature of hypothalamic-pituitary-adrenal (HPA) activity, characterized by a cortisol surge that peaks around 30–45 mins after awakening [2]. Contemporary viewpoints position the CAR as a proxy for baseline stress [3], reflecting both prior stress experienced and stress anticipation. As supporting evidence, a larger CAR emerged on the morning of shooting competition (vs. training) [4], archery elimination (vs. qualification) [5], and the pre-ascent phase of climbing (vs. resting day) [6]. Others have reported no CAR changes across a sporting competition [7, 8]. Further discrepancies were documented in a review of exercise and CAR studies with training factors (e.g., load acclimatization, intensity thresholds) identified as confounders [1].

Skeletal muscle damage offers an alternative explanation for divergent sport results. Support comes from research comparing athletes with the overtraining syndrome (OTS) and healthy athletes [9]. Upon initial diagnosis, the OTS group presented a flatter CAR and a higher creatine kinase (CK) level than the healthy group, indicating greater muscle trauma and damage. After three months recovery, the OTS group presented a CAR and CK profile that was comparable to healthy athletes. Similarly, triathletes (N = 2) diagnosed with the OTS had a lower CAR at the end of the season, relative to the season start [10]. Conversely, non-overtrained athletes showed a progressive rise in the CAR across the season [10]. Such findings suggest a link between the inflammatory response to excessive training loads, among other factors (e.g., insufficient recovery), and post-awakening cortisol secretion.

Cortisol regulation of human inflammation is well recognized [11, 12] and offers a theoretical model to explain CK and CAR dynamics in sport. As a glucocorticoid, cortisol exerts both pro- and anti-inflammatory effects on immune and inflammatory reactions following a systemic stimulus [11, 12]. The bi-directional flow of information between the immune and neuroendocrine systems to repair damaged tissue and maintain homeostasis [12] could involve shifts in early-morning cortisol secretion. Regeneration of damaged muscle tissue might also promote secondary changes in cortisol secretion, due to metabolic factors related to control and recovery [13, 14]. To our knowledge, the impact of training on the CAR and muscle damage, as a mediating factor representing exercise-induced inflammation, has yet to be explored. A comparison of CAR and CK adaptations across training blocks that differ in load intensity (i.e., heavy vs. light) would be a logical first step.

The hypothalamic-pituitary-gonadal (HPG) axis, and its end product in male’s testosterone, also adapts to training and competition stress [13]. Like cortisol, testosterone exhibits an awakening response (TAR) but is typified by a concentration decline over the same post-awakening period [15, 16]. Studies on rugby players and climbers report equivocal TAR results in terms of activity profiles and training adaptation [6, 17, 18]. Such inconsistencies could reflect more complex HPA- and HPG-axes interplay, whereby cortisol and testosterone reciprocally interact with upstream regulatory centers at each axis level [19, 20]. Partial support comes from positive CAR and TAR associations in healthy men [15, 16] and suggests that individuals presenting a larger CAR also tend to possess a flatter TAR. It remains unclear if, and how, muscle damage directly affects the TAR or whether sport-related TAR changes are driven by cortisol. Experimental testing of both hormonal features, and ensuing relationships with each other and CK, would help answer these questions.

This study investigated the CAR, TAR, and muscle damage in elite male judokas following light and heavy training weeks. Judo is a sport linked to varying degrees of muscle damage and hormonal change with different exercise and training configurations [21, 22, 23, 24, 25, 26]. Therefore, the targeting of male judokas would provide an ideal model for this training-based study. Our aims were to; (1) examine the impact of training at different intensities on reactive- and volume-based CAR and TAR metrics, as well as CK, and (2) evaluate CK as a causal mediator of training differences in the CAR and TAR. As a secondary aim, we explored our dataset for positive CAR and TAR associations. In relation to these aims, we first hypothesized that heavy training would suppress the CAR and TAR, and elicit more muscle damage (i.e., higher CK level) versus light training. Our second hypothesis was that the CAR and TAR differences would be mediated by CK activity. Finally, we anticipated that positive CARTAR linkages would emerge.

## MATERIALS AND METHODS

### Participants

Twenty elite male judokas (mean age = 24.4, SD = 2.0 years, mean weight = 80.4, SD = 10.5 kg) were recruited for this study. The participants had no current injuries, nor any medical or health conditions, that would affect their ability to complete the research procedures. Written informed consent was given before the study commenced, with approval from the ethics committee at the Institute of Sport – National Research Institute, Warsaw. This research was implemented in a preparatory training camp held at the Central Sport Centre, Zakopane.

### Study design

A quasi-experimental, single-group, post-only design was used to address the study hypotheses. In our cohort of elite male judokas, CAR, TAR, and CK measures were taken after two consecutive training weeks that differed in session intensity and subsequently compared. In week one, lighter sessions were prescribed over six consecutive days of judo training (Monday to Saturday), followed by a recovery day (Sunday) before testing (Monday). The delayed assessment (~2 days after the last training session) removed the effect of acute exercise on next-day CAR [27, 28]. These procedures were replicated in week two, but a heavier workload was prescribed in each training session, as described in more detail below. The participants were housed together at the Central Sport Centre throughout this study. This ensured that dietary selection, meal timing, training activities, bedtime and time of awakening were consistent across athletes.

### Training protocols

The participants completed 9–10 training sessions (60–90 mins each) each week aimed at enhancing technical performance, whilst developing general and sport-specific fitness. These goals were achieved via different exercising configurations; (1) sessions focusing on judo throwing and grappling techniques (e.g., ippon seoi nage, katame waza), (2) simulated judo bouts (i.e., randori) between opponents of a similar size and experience, each lasting 5 mins with a 5-min rest interval between bouts; (3) circuit training consisting of repeated bouts of 30-sec efforts (e.g., skipping, push-ups, sit-ups, jump squats, 20 m shuttle runs, burpees), and (4) fitness examinations using the Special Judo Fitness test [29], which lasts 75 sec and produces an elevated heart rate (up to 190 bpm) and blood lactate (up to 15 mmol/L) response. Heavy training was differentiated from light training in that more throws and grappling activities per technical session, randori per session (4 vs. 2), repeated efforts (3–4 vs. 2) during circuit training, and fitness tests (2 vs. 1) were performed. Rest intervals between sets and exercises were consistent throughout both training blocks. All sessions began with a standard warm-up comprising of basic throws (i.e., uchikomi) and dynamic stretching.

### Morning assessment

Following an overnight (> 8 hours) fast, capillary blood samples were collected at three post-awakening points (+3, +30, +60 mins) for CAR and TAR determination. This 3-point protocol was chosen to capture the peak awakening surge (~30–45 mins) in cortisol [2, 30]. Additionally, the frequency and duration of sampling was deemed achievable in a field setting, without over-burdening the athletes involved. Creatine kinase was measured only once (+3 mins). The participants reporting waking under alarm conditions in each weekly assessment, between the hours of 6.20 am and 7.00 am. To remove the effect of dietary intake [30], no food or drinks were taken until all blood samples were collected. Participants were instructed to rest (in a seated position) during the 60-min sampling period, thereby eliminating any hormonal bias due to physical movement.

Capillary blood samples (~300 μL at each time point) were collected by trained technicians into heparinized tubes. After sample centrifugation, the plasma portion was transferred into polypropylene tubes for storage at -80 °C. The recruited athletes were routinely tested for capillary blood biomarkers and thus, were less likely to exhibit a stress response due to the sampling procedure. Anecdotally, there is much less discomfort with capillary blood collections as opposed to venipuncture [31]. Cortisol measured in capillary blood also tracks well, and correlates strongly (*r* = 0.89) with, venous blood during acute stress induction [31]. As a further benefit for CAR research, capillary or venous blood measures can better capture the timing of cortisol peaks due to blood-saliva lag delays [27, 31, 32]. The same reasoning applies to TAR measurements.

Plasma cortisol and testosterone concentrations were determined using enzyme-linked immunoassay kits (DRG, Germany). The cortisol and testosterone kits had a sensitivity limit of 1.3 ng/mL and 0.083 ng/mL, respectively. Inter-assay variability on kit-supplied controls, expressed as a coefficient of variation, did not exceed 4%. All participants’ samples were assayed in the same plate to eliminate inter-assay variation in the studied hormones. Plasma CK level, expressed in U/L, was determined spectrophotometrically using a biochemical analyzer (Biotecnica Instruments, Rome, Italy) and reagents provided by the manufacturer.

### CAR and TAR features

Two complementary metrics were chosen to index the CAR and TAR; (1) change scores that reflect hormone reactivity and (2) area under the curve (AUC) measurements representing hormone secretory volume. To overcome interpretive issues arising from basal hormonal differences (e.g., due to sports training), both metrics were corrected for baseline values. Change scores were calculated as the raw difference at the 30-min (i.e., T2 [+30 mins] – T1 [+3 mins]) and 60-min (i.e., T3 [+60 mins] – T1 [+3 mins]) time points and expressed using the following notation: CAR_Δb30_, CAR_Δb60_, TAR_Δb30_, and TAR_Δb60_. The AUC for the 30-min and 60-min sampling periods was determined using a trapezoidal method [4, 27, 33] and expressed as follows: CAR_AUCb30_, CAR_AUCb60_, TAR_AUCb30_, and TAR_AUCb60_.

In the absence of a control group, reference blood cortisol and testosterone data were obtained from healthy men (N = 14–16) participating in a CAR study [34] and sleep research [35]. Study means were extracted from the article figures using WebPlotDigitizer (https://apps.automeris.io/wpd/). Reference values for blood CK were extracted from research on male judokas [23, 24] and collapsed into a single dataset (N = 23). The extracted means only served as a reference point and were not analyzed statistically.

### Data analyses

The study data were analyzed using R software [36]. As a preliminary step to detect any post-awakening trends, the original cortisol and testosterone time series were plotted using local polynomial regression with a 95% confidence interval (CI). The reference datasets were also plotted, but no CI was estimated as individual data points were not available. Next, bivariate relationships between the CAR, TAR, and CK measures were explored using Pearson correlations (*r*). These correlations, computed after pooling data across both study weeks, were classified using the standard conventions of a weak (*r* = 0.20 to < 0.40), moderate (*r* = 0.40 to < 0.60), strong (*r* = 0.60 to < 0.80) or very strong (*r* ≥ 0.80) effect.

To identify any training effect on the studied variables, each CAR, TAR, and CK metric was tested using a one-way, repeated measures analysis of variance. Models were constructed in the lmerTest package [37] using the linear mixed-effects procedure. Our specifications included training condition (light, heavy), as a within-subjects factor, and random intercepts for each participant. Checks of model residuals revealed that all normality assumptions were met. Results are plotted as estimated marginal means with a 95% CI. As an effect-size statistic, Cohen’s *d* score was computed for each comparison and classified as a small (*d* = 0.20 to < 0.50), medium (*d* = 0.50 to < 0.80), large (*d* = 0.80 to < 1.20) or very large (*d* = 1.20+) effect.

To test whether CK acts as an intervening factor between training condition and the CAR or TAR, a series of causal mediation models were performed [38]. After standardizing all continuous variables, we first defined two effects using linear regression (i.e., training → mediator, training → outcome). Both outputs were entered into the mediation package [38] to estimate three pathways: (1) *direct effect* of training condition on each dependent variable (absent the mediator), (2) *indirect effect* of training condition on each dependent variable (going through the mediator), and (3) *total effect* representing the sum of direct and indirect effects. Estimates are provided with a bootstrapped 95% CI using a Quasi-Bayesian method (N = 5000 simulations). Statistical significance was set at an alpha level of *p* ≤ 0.05.

## RESULTS

All the plasma CAR parameters were strongly, and positively, interrelated with each other (*p* < 0.001, Table 1). The most prominent relationships, ranging from very strong to perfect (*r* = 0.96–1.00), emerged between the CAR_Δb30_, CAR_AUCb30_, and CAR_AUCb60_. Likewise, the plasma TAR measures were strongly and positively related (*p* < 0.001), especially the TAR_Δb30_, TAR_AUCb30_, and TAR_AUCb60_ (*r* = 0.95–1.00). The plasma CAR and TAR metrics were all unrelated to each other (*p* > 0.154). Some trending (*p* = 0.062–0.063) negative, but weak, relationships were seen between plasma CK concentration and several CAR measures (i.e., CAR_Δb30_, CAR_AUCb30_, CAR_AUCb60_). The plasma CAR_Δb60_ and all TAR metric associations with CK concentration were found to be non-significant (*p* > 0.146).

**TABLE 1:** Pearson correlations between the plasma reactive changes and area under the curve measures of cortisol and testosterone, and creatine kinase (CK) concentration. Correlations are presented with a 95% CI in brackets.

Parameter	CAR_Δb60_	CAR_AUCb30_	CAR_AUCb60_	TAR_Δb30_	TAR_Δb60_	TAR_AUCb30_	TAR_AUCb60_	CK
CAR_Δb30_	0.68^#^	1.00^#^	0.96^#^	-0.19	-0.18	-0.19	-0.21	-0.30
	(0.47, 0.82)	(1.00, 1.00)	(0.92, 0.98)	(-0.47, 0.13)	(-0.47, 0.13)	(-0.47, 0.13)	(-0.49, 0.11)	(-0.56, 0.02)
CAR_Δb60_		0.68^#^	0.87^#^	-0.19	-0.22	-0.19	-0.22	-0.23
		(0.47, 0.82)	(0.76, 0.93)	(-0.47, 0.13)	(-0.50, 0.10)	(-0.47, 0.13)	(-0.50, 0.10)	(-0.51, 0.08)
CAR_AUCb30_			0.96^#^	-0.19	-0.18	-0.19	-0.21	-0.30
				(-0.47, 0.13)	(-0.47, 0.13)	(-0.47, 0.13)	(-0.49, 0.11)	(-0.56, 0.02)
CAR_AUCb60_				-0.20	-0.22	-0.20	-0.23	-0.30
				(-0.48, 0.12)	(-0.49, 0.10)	(-0.48, 0.12)	(-0.50, 0.09)	(-0.56, 0.02)
TAR_Δb30_					0.60^#^	1.00^#^	0.95^#^	0.13
					(0.35, 0.76)	(1.00, 1.00)	(0.91, 0.97)	(-0.19, 0.42)
TAR_Δb60_						0.60^#^	0.81^#^	0.14
						(0.35, 0.76)	(0.67, 0.90)	(-0.18, 0.44)
TARAUCb_30_							0.95^#^	0.13
							(0.91, 0.97)	(-0.19, 0.42)
TAR_AUCb60_								0.15
								(-0.17, 0.44)

Significant correlation at ^#^*p* < 0.001.

**FIG. 1:**
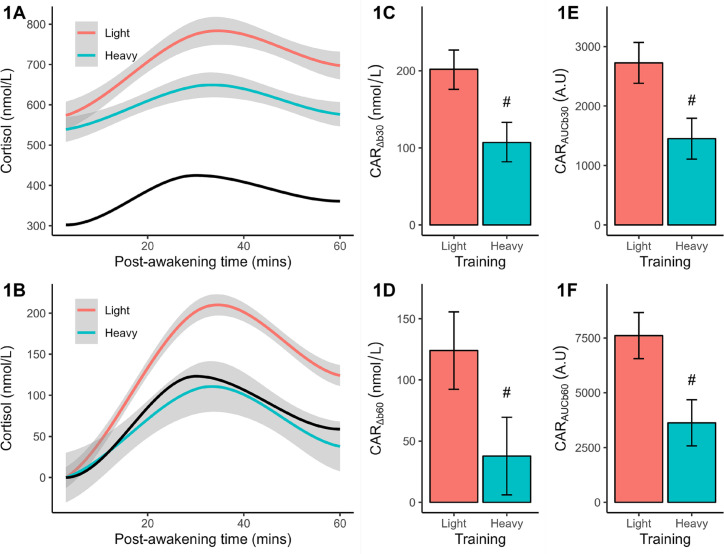
Plasma measures of cortisol concentration (original, baseline-corrected), reactive changes (CAR_Δb30_, CAR_Δb60_), and area under the curve (CAR_AUCb30_, CAR_AUCb60_) following light and heavy training weeks. The smoothed concentration data are presented with a 95% CI. The solid black line indicates the smoothed reference data (means only). All CAR results are shown as marginal means with a 95% CI. Significant from light training #*p* < 0.001.

Analysis of baseline (+3 mins) cortisol revealed no significant (*p* = 0.087) difference between light (marginal mean = 574 nmol/L) and heavy (marginal mean = 539 nmol/L) training, but the effectsize difference was large (*d* = 0.83) and potential meaningful. Qualitatively, both responses exceeded the reference group mean of 302 nmol/L. In the light training condition (Figure 1A), cortisol increased after waking and peaked after ~30 mins before declining; a pattern mirrored by heavy training and reference data, but at lower concentrations across most (or all) time points. The CAR shape remained once plotted after baseline corrections (Figure 1B), but training and reference differences were magnified. Statistical comparisons between training conditions verified a larger (*p* < 0.001) CAR_Δb3__0_ (Figure 1C), CAR_Δb6__0_ (Figure 1D), CAR_AUCb30_ (Figure 1E), and CAR_AUCb60_ (Figure 1F) after light than heavy training. The effectsize differences ranged from 2.14 to 2.64 or very large effects.

**FIG. 2:**
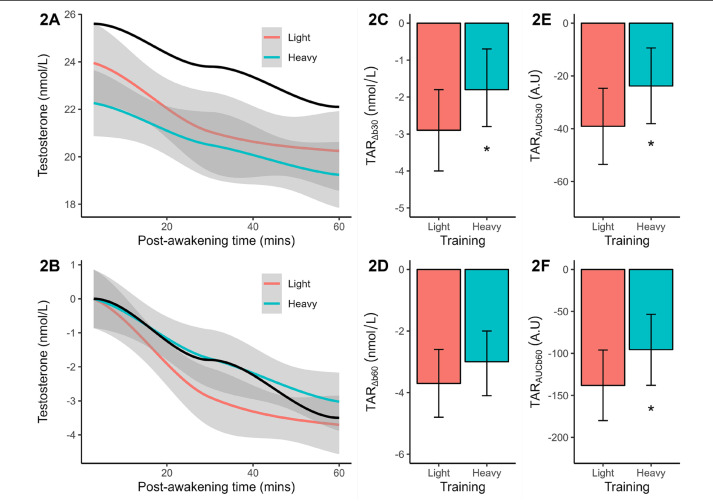
Plasma measures of testosterone concentration (original, baseline-corrected), reactive changes (TAR_Δb30_, TAR_Δb60_), and area under the curve (TAR_AUCb30_, TAR_AUCb60_) following light and heavy training weeks. The smoothed concentration data are presented with a 95% CI. The solid black line indicates the smoothed reference data (means only). All TAR results are shown as marginal means with a 95% CI. Significant from light training ^*^p ≤ 0.05.

Baseline testosterone concentration differed significantly (*p* = 0.038, *d* = 1.02 or large effect) between light (marginal mean = 23.9 nmol/L) and heavy training (marginal mean = 22.3 nmol/L). Both results were slightly lower than the reference mean of 25.6 nmol/L. Post-awakening testosterone decreased with similar slopes in both training weeks and reference data (Figure 2A). After baseline corrections, the post-awakening decline in testosterone concentration was found to be less pronounced after heavy (vs. light) training (Figure 2B). Significance testing of the TAR_Δb30_ (*p* = 0.051, Figure 2C), TAR_AUCb30_ (*p* = 0.051, Figure 2E), and TAR_AUCb60_ (*p* = 0.035, Figure 2F) confirmed a flatter early-morning trajectory after heavy training, compared to light training, with large effect-size differences (*d* = -0.95 to -1.04). No significant training effect on the TAR_Δb60_ was identified (Figure 2D).

**FIG. 3:**
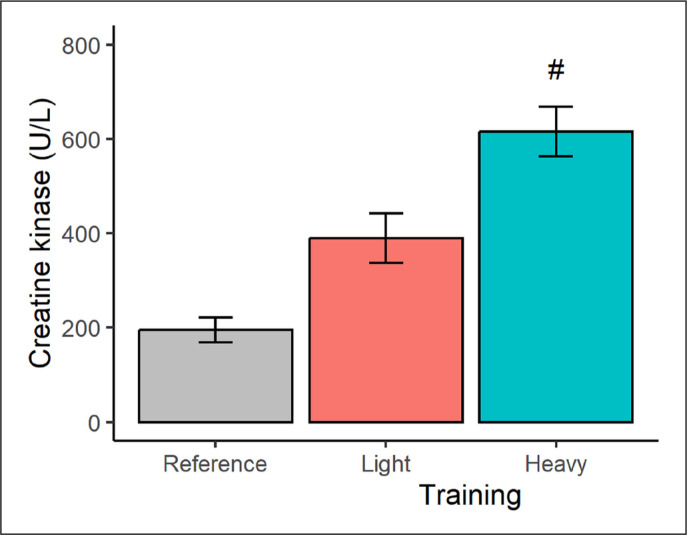
Plasma creatine kinase concentration in male judokas following light and heavy training weeks. Results are presented as marginal means with a 95% CI. Reference data are shown as a descriptive mean with a 95% CI. Significant from light training #*p* < 0.001.

In terms of muscle damage (Figure 3), a higher (*p* < 0.001) CK concentration was seen after the heavy (vs. light) training condition, representing a very large effect-size difference of 4.70. The CK marginal means after the light and heavy training weeks also exceeded, at least qualitatively, the CK reference data by 100% and 216%, respectively.

**FIG. 4:**
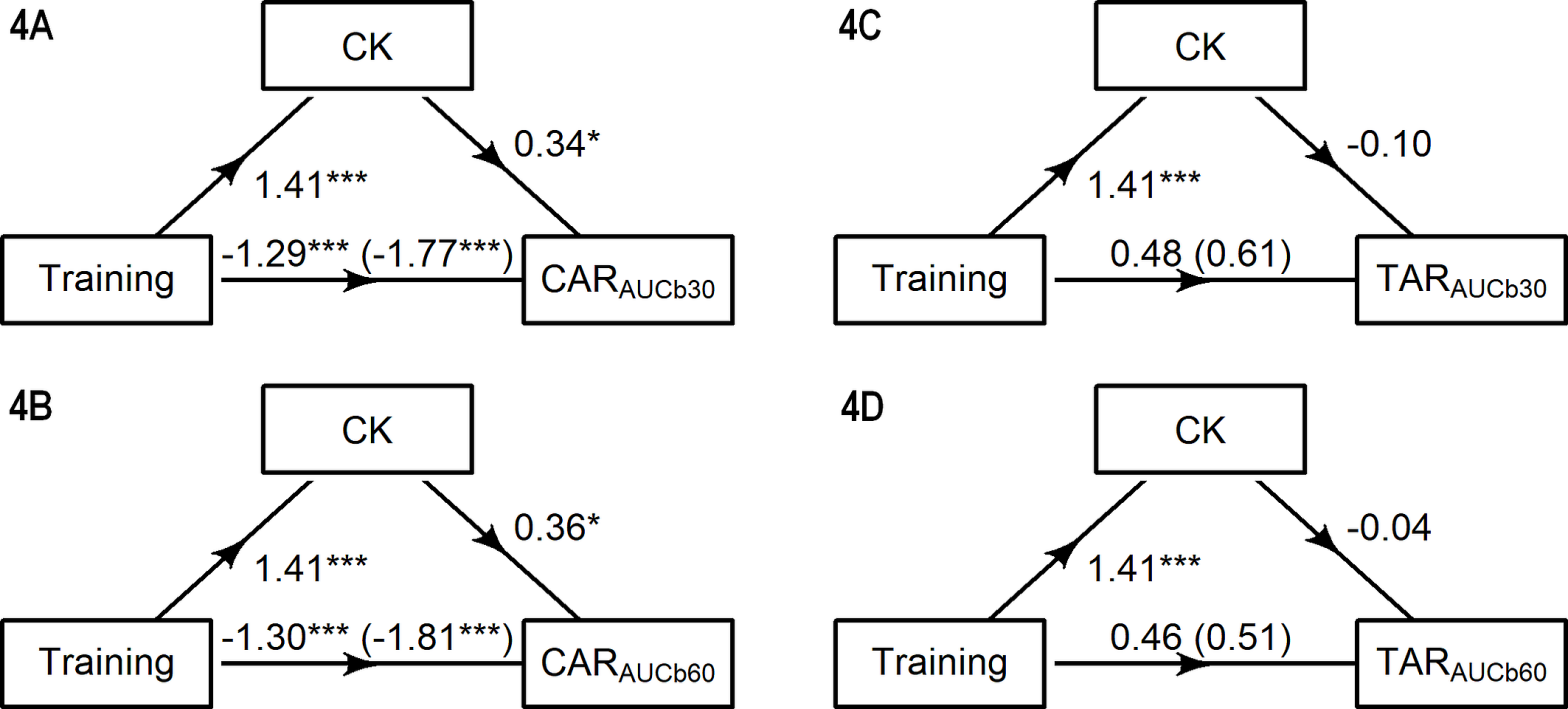
Path models showing the effect of training condition on the CAR and TAR measures, as mediated by creatine kinase (CK). Total effect (path c) represents the effect of training on CAR_AUCb30_, CAR_AUCb60_, TAR_AUCb30_ and TAR_AUCb60_ with no mediator. Direct effect (path c’) represents the effect of training on each outcome when the mediator is included in the model. Indirect effects (paths a*b) represent the effect of training on CAR_AUCb30_, CAR_AUCb60_, TAR_AUCb30_ and TAR_AUCb60_ through CK. Each figure shows the standardized regression coefficients (*p < 0.05, ***p < 0.001).

The mediation results are plotted in Figure 4. Only the AUC outputs are presented to eliminate redundancies arising from strongly or perfectly correlated variables. Mediation analyses yielded a total and direct effect of training condition (*p* < 0.001) on the CAR_AUCb30_ (Figure 4A) and CAR_AUCb60_ (Figure 4B). The indirect CK-mediated effect of training on the CAR_AUCb30_ (estimate = 0.48, 95% CI 0.06, 0.17) and CAR_AUCb60_ (estimate = 0.50, 95% CI 0.03, 1.06) were also significant (*p* ≤ 0.040). The total and direct effects of training on the TAR_AUCb30_ (Figure 4C) and TAR_AUCb60_ (Figure 4D) were nonsignificant. Likewise, the indirect effects of CK activity on the TAR_AUCb30_ (estimate = -0.14, 95% CI -0.63, 0.64) and TAR_AUCb60_ (estimate = -0.06, 95% CI -0.53, 0.64) were not significant.

In summary, the CAR to training was partly mediated by CK, whilst no mediation effect on the TAR emerged.

## DISCUSSION

This study investigated the impact of short training blocks at different intensities on plasma CAR, TAR and skeletal muscle damage (indexed by CK) in elite male judokas, and the intervening effect of muscle damage on the CAR and TAR. As hypothesized, the earlymorning surge in cortisol concentration was reduced after a heavy (vs. light) training week. Heavy training also attenuated the postawakening decline in testosterone concentration, and enhanced CK release, compared to light training. Aligning to our second hypothesis, the CK training response partly mediated the CAR, but not TAR, differences.

After the light training block, plasma cortisol values rose by 36% (+30 mins) and 22% (+60 mins) over waking levels, whilst plasma testosterone values decreased by -11% and -15% at these time points, consistent with general findings in athletic [4, 5, 7, 8, 17, 18, 33] and non-athletic men [2, 15, 16, 34, 39]. Training intensity appears to play a regulatory role with a heavy training block suppressing the CAR (21% and 8% increases) and attenuating the TAR (-7% and -13% decreases), respectively. Parallel results come from male rugby players, who displayed a smaller CAR and/or a flatter TAR across a short training camp [17, 18] that could reflect higher workloads and physiological stress. It is notable that the plasma CAR to training, and reference data, are smaller in magnitude than saliva-based hormone research, where CAR increases of up to 50–120% have been described [2, 15, 27, 34]. Comparative studies show that the salivary CAR (as a %) is more than double the blood CAR [27, 34].

The training-induced CAR seen in male rugby players [17, 18] and male judokas differs from other sporting groups. For instance, a higher training load was linked to a larger CAR (delta and % change) in recreational runners, but no association was identified for cortisol AUC measurements [40]. Similarly, a 7-day period of intensified training in male footballers increased awakening and AUC cortisol concentrations, but the rate of cortisol increases and peaks did not change [33]. The physicality and physiology of each sport could explain these differences. Both judo and rugby are contact sports with high anaerobic demands, whilst running and football are non-contact sports with high aerobic (at least for running) demands. This lack of consistency across studies underscores the need for a mechanistic approach that targets key drivers and intervening factors, which might help contextualize any reported discrepancies.

In judokas, different exercise and training session configurations can elicit muscle damage of varying degrees [21, 22, 23, 24]. Here, a short block of heavy training promoted a dramatic rise in morning CK level from light training and both responses exceeded reference CK in a dose-response manner (light by 100%, heavy by 216%). The time course of muscle damage has implications for this study. For instance, three days of judo-specific training increased CK level, from a resting value of 211 U/L up to 795 U/L immediately post the last training session, before declining to 648 U/L some 12 hours later [23]. This pattern suggests an underestimation of muscle injury in this work, since CK was assessed nearly two days after the preceding workout. Nonetheless, the inflammatory response to muscle injury is a complex process, activating a myriad of proand anti-inflammatory cytokines, proteins, and oxidative stress markers on different time scales [22, 23, 24]. Hence, for a more detailed inspection of this process, a broader battery of biochemical markers with more frequent post-exercise or post-training sampling is recommended.

Our principal finding was that muscle damage mediated, in part, differences in the plasma CAR. Parallel results come from research on OTS athletes, who presented a lower CAR and higher CK level than healthy athletes at initial testing, but similar CAR and CK profiles after three months recovery [9]. Other descriptive and experimental work in sport suggest a CAR link to muscle damage [10, 17, 41]. Speculatively, the mechanism/s behind these linkages exist in the anti- and pro-inflammatory effects of cortisol [11, 12], which coexist and are imbedded in complex dynamics with the ascending and descending circadian phases [12]. As cortisol moves towards its ascending early-morning peak, suppressive effects rise and dominate over permissive effects [12]. Accordingly, when recovering from a prior inflammatory stimulus (e.g., heavy training), a smaller CAR might be adaptive by ensuring that permissive effects dominate to mount an efficient defensive response to imminent stressors. By this logic, a larger CAR seen under normal (e.g., light training) conditions could be more suppressive, but still adaptive, to meet homeostatic needs. These ideas await experimental testing in sport, as does a potential metabolic role for the CAR [13, 14].

The induction of muscle damage did not, however, mediate the plasma TAR. As one explanation, much smaller standardized effects on the TAR (< 1.1) than CAR (> 2.1) materialized. Baseline testosterone secretion also exhibits greater sensitivity to sports training than cortisol [13], as we illustrated, and is a major determinant of testosterone’s post-awakening trajectory. Supporting this, initial (+3 min) testosterone concentration was strongly, and negatively, related to all TAR metrics (*r* = -0.75 to -0.83, *p* < 0.001) corrected for baseline differences. Other potential explanations exist, given that intra-individual variability in the daily rate of testosterone change is primarily due to individual-specific environmental factors [39]. This may include, but not limited to, speculated TAR roles to help partition testosterone’s primary functions and target tissues to muscle anabolism while asleep, and to catabolism, behavior and social interaction while awake [16].

Contrary to initial expectations, we did not find any positive CAR and TAR associations. These null findings do come with important caveats. First, only between-subject CAR and TAR relationships were tested, not within-person associations where cortisol and testosterone linkages across the day often reside [20, 42, 43]. Second, stronger time-lagged associations (up to 60 mins) between cortisol and testosterone have been reported [20, 42] that could not be evaluated under the current sampling regime. Third, CAR modulation appears to be partly dependent on nocturnal HPA activity [34], driven by regulatory inputs (e.g., adrenocorticotropin hormone) beyond those psychophysiological processes specific to the sleep-wake transition. The same principle applies to those HPG signals (e.g., luteinizing hormone) that govern testosterone production. Therefore, a dedicated analysis of these HPA and HPG signals, across the late nocturnal and post-awakening periods, is needed to explicate interaxes coordination at this critical junction.

Several practical applications emanate from this work. Conceptualizing muscle damage as an intervening variable could explain the lack of CAR shifts in some competitive sports [7, 8], due to existing injuries and inflammation. It also offers a mechanism to explain a depressed CAR following a late evening exercise session [27] and rising CAR prior to challenging events [4, 6], since prior exercise intensity and/or volume is typically reduced. Awareness of impending muscle damage (e.g., after intensified training) might also guide novel interventional strategies. One being early-morning light exposure to boost the CAR [44] until inflammation subsides. The observed CAR and TAR adaptations could further assist study planning. Training intensity, for instance, can be adjusted to manipulate post-awakening hormones and test other hypotheses. From a measurement perspective, we discovered that some CAR and TAR metrics (i.e., Δb30, AUCb30, AUCb60) are interchangeable, due to very strong or perfect *r* values. Likewise, the emergence of different effect sizes (CK > CAR > TAR) and thus, sensitivities to the same training stimuli, can inform instrument selection in sports research and practice.

The study strengths include the ecological design, collection of blood samples, and extended (up to 60 mins) post-awakening sampling period. Nevertheless, this work does have inherent limitations. Statistical power was compromised by the small sample and postonly assessment design. The targeting of male judokas also limits the generalizability of our findings to non-combat sport athletes and sedentary populations. Furthermore, we were unable to counterbalance the training interventions, due to practical constraints when working with elite athletes. On the other hand, any cross-over effect is minimized by the scheduling of light training first. Finally, we did not quantify individual workloads at each training intensity, although the CK results infer different physiological and physical demands, as does training and non-training (i.e., reference) comparisons. Still, this information would be a useful adjunct in future work to establish load thresholds for the CAR and TAR, and to investigate individual dose-response variability in these outcomes [1, 5, 40].

## CONCLUSIONS

The CAR and TAR are distinct hormonal features with emerging focus in sport, but lacking a strong mechanistic understanding. This study on elite male judokas revealed a suppressed CAR, and attenuated TAR, following a short block of heavy versus light training. Differential CK release patterns (heavy > light training), as a proxy for skeletal muscle damage, partly explained the CAR differences. Viewing muscle damage as an intervening factor could therefore inform the design and interpretation of hormone-awakening research.

## Data Availability

The research data collected are unavailable due to confidentiality agreements.
